# Large-Scale and Defect-Free Silicon Metamaterials with Magnetic Response

**DOI:** 10.1038/srep25760

**Published:** 2016-05-19

**Authors:** Ningbo Yi, Shang Sun, Yisheng Gao, Kaiyang Wang, Zhiyuan Gu, Siwu Sun, Qinghai Song, Shumin Xiao

**Affiliations:** 1 Integrated Nanoscience Lab, Department of Material Science and Engineering, Shenzhen Graduate School, Harbin Institute of Technology, Shenzhen, 518055, China; 2 Integrated Nanoscience Lab, Department of Electronic and Information Engineering, Shenzhen Graduate School, Harbin Institute of Technology, Shenzhen, 518055, China

## Abstract

All-dielectric metamaterials offer a potential low-loss alternative to plasmonic metamaterials at optical frequencies. Here, we experimentally demonstrate a silicon based large-scale magnetic metamaterial, which is fabricated with standard photolithography and conventional reactive ion etching process. The periodically arrayed silicon sub-wavelength structures possess electric and magnetic responses with low loss in mid-infrared wavelength range. We investigate the electric and magnetic resonances dependencies on the structural parameters and demonstrate the possibility of obtaining strong dielectric-based magnetic resonance through a broad band range. The optical responses are quite uniform over a large area about 2 × 2 cm^2^. The scalability of this design and compatibility fabrication method with highly developed semiconductor devices process could lead to new avenues of manipulating light for low-loss, large-area and real integrated photonic applications.

Metamaterials, the artificial electromagnetic sub-wavelength structures with unique properties, have yielded many exciting optical phenomena including super-resolution imaging^1^,^2^,^3^, invisibility cloaking^4^,^5^,^6^, and perfect absorption^7^. The basic requirement of metamaterials is to exhibit artificial optical magnetism using sub-wavelength metallic nanostructures. Despite rapid advances in this field, metamaterials at optical frequencies have often proven to be impractical due to the significant loss from the metallic resonators^8^. Although gain compensation has emerged as a promising strategy to avoid the deleterious impacts of losses on plasmonic metamaterials, it is almost impossible to fabricate active metamaterial in large area or three-dimension (3D)^9^. The recent development of high refractive index dielectric nanostructure offers an alternative solution to the material loss^10^. Due to the Mie resonances, a number of dielectric nanostructures such as nanoparticles, nanowires and nanoblocks have been found to exhibit strong magnetic and electric resonances^11^,^12^,^13^,^14^. Proper control of lattice arrangement, resonator geometry, and composition materials allows the adjustments of effective permittivity and permeability of dielectric metamaterial. Because of the absence of ohmic loss, dielectric metamaterials have shown much smaller absorptive than their metallic counterparts. And their simple unit-cell geometries offer the possibility to achieve three-dimensional and isotropic metamaterials^15^.

The implementations of dielectric metamaterials have so far been experimentally characterized in a broad wavelength range. However, specified in mid-infrared wavelength range, the dielectric materials are limited in tellurium (Te) and germanium (Ge) at mid-infrared wavelengths^16^,^17^,^18^. Compared with Te and Ge, silicon (Si) has relatively lower index of refraction. Thus the performances of Si based magnetic resonators have been considered to be not as good as Te and Ge devices in mid-infrared^13^,^19^,^20^. As we know, as silicon holds a preeminent position in photonics communication in the past 40 years, the fabrication procedure of silicon based devices have been developed to a fully-fledged level. On the contrary, the fabrication technique of Te and Ge are much less mature than that of silicon. Therefore, considering the practical applications of dielectric based metamaterials, it is highly desirable to develop silicon based magnetic metamaterial in mid-infrared wavelength, especially for the large-scale devices^21^,^22^,^23^.

Furth more, the sizes of basic unit cells of dielectric metamaterials are usually in sub-wavelength scale. This kind of small features can be precisely fabricated using focused-ion-beam milling (FIB) or electron-beam lithography (EBL). But the low throughput of these techniques is not suitable for fabricating a metamaterial device with size as large as centimeter scale which is typical required for real applications. In order to develop a scalable scheme that enables the large-area fabrication of 3D nanostructures, nanofabrication method with high-throughput capability, such as nanoimprinting and nanosphere lithography, have been proposed and demonstrated to produce large scale silicon metamaterials in the visible and near-IR spectral range^24^,^25^,^26^,^27^. However, these methods suffer a lot from defects over large fabrication area and are not well suitable for fabricating structures with multiple layers, where precise alignment is usually required.

In this paper, we explore the possibility to obtain Si based dielectric metamaterial with both of electric and magnetic responses in mid-infrared wavelength range. By designing the structure of Si sub-wavelength structure with sufficiently small array spacing, the natural shortcoming of lower refractive index comparing to Te and Ge has been overcome. Large-scale and defect free silicon magnetic metamaterial with size up to 2 × 2 cm^2^ has been experimentally realized for the first time. From the experimental results, magnetic resonance is obtained through a very broad bandwidth from 7.4 ~ 8.3 μm. The Si based metamaterials is fabricated using standard photolithography combined with typical reactive ion etching, promising the realization of wafer-scale low-loss dielectric metamaterial in practical applications.

## Results and Discussions

**Figure 1:**
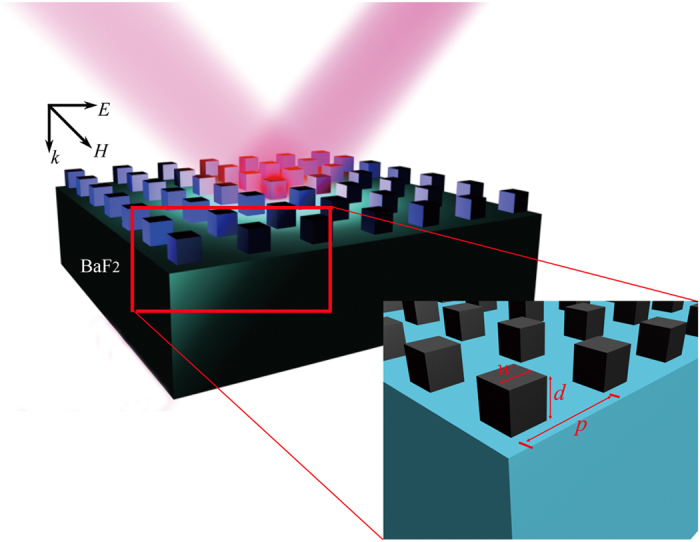
Illustration of the structure and optical response in a lattice of Si cuboid unit cells with height d and length w. The periodicity of the lattice is *p*. The IR incident beam illuminates from the front side of periodic Si cuboid blocks. Exciting Mie type electric and magnetic dipolar resonances with controlled spectral positions will create electric and magnetic resonances in the near field of the structures.

The schematic picture of the designed silicon dielectric magnetic metamaterial is shown in Fig. 1. It is composed with a periodic lattice made of a Si cuboid resonator on top of Barium fluoride (BaF_2_) with refractive index equal to 1.471. The relative high dielectric constant of Si localizes the electromagnetic wave on a scale much shorter than the wavelength in free space. Thus various resonances, which correspond either to an electric dipole or to an artificial magnetic polarization, can be imagined. To investigate the magnetic and electric activities, Si sub-wavelength cuboid arrays are studied under polarized incident light with commercial Finite Element Method (FEM) simulation software, COMSOL Multiphysics. The simulation details can be found in the Methods. The refractive index of Si is set as 3.427 + 0.02*i in consideration of experimental roughness of real fabricated structures and according to the measurement result measured by fitting transmission analysis on a homogeneous Si thin film^28^, and the width of the cuboid in the cross-section ranges from 1.2 to 3 μm, which could work as an optical scatter based on Mie theory.

**Figure 2:**
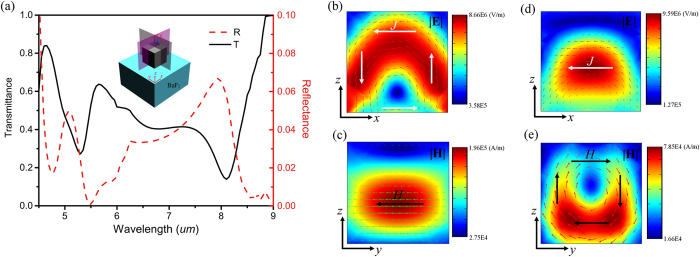
(**a**) The transmission/reflection/absorption spectra of the Si cuboid arrays. (**b**) The electric field distribution in x-z plane with y = 0 at *λ*_M_  = 8.06 μm inside the cuboid, with the white lines and red arrow indicating displacement current. (**c**) The magnetic field distribution in y-z plane with x = 0 at *λ*_M_ = 8.06 μm inside the resonator, with the black and green arrows indicating the magnetic field direction. (**d**) The electric field distribution in x-z plane with y = 0 at *λ*_E_ = 6.46 μm inside the resonator, with the red and white lines and arrow indicating displacement current. (**e**) The magnetic field distribution in y-z plane with x = 0 at *λ*_E_ = 6.46 μm inside the Si cuboid, with the black and green arrows indicating the magnetic field direction.

The reflection and transmission spectra are calculated in the far-field at x-y planes centered 38 μm away from top and bottom surfaces of the structure, respectively. The height d and the period p of the Si cuboid are kept as 1.6 μm and 3.2 μm, respectively. And the width w is selective as 2.2 μm. The calculated transition and reflection spectra are presented in Fig. 2(a), showing a strong dependence on the incident wavelength. We can easily see two resonances near 8.06 μm and 6.46 μm, which correspond to the peaks in the reflection spectrum and the minima in the transmission spectrum with only 10% of the incident energy transmitted through the structure at 8.06 μm. For the wavelengths off the resonances, almost of incident electromagnetic energy is transmitted through the arrays.

**Figure 3:**
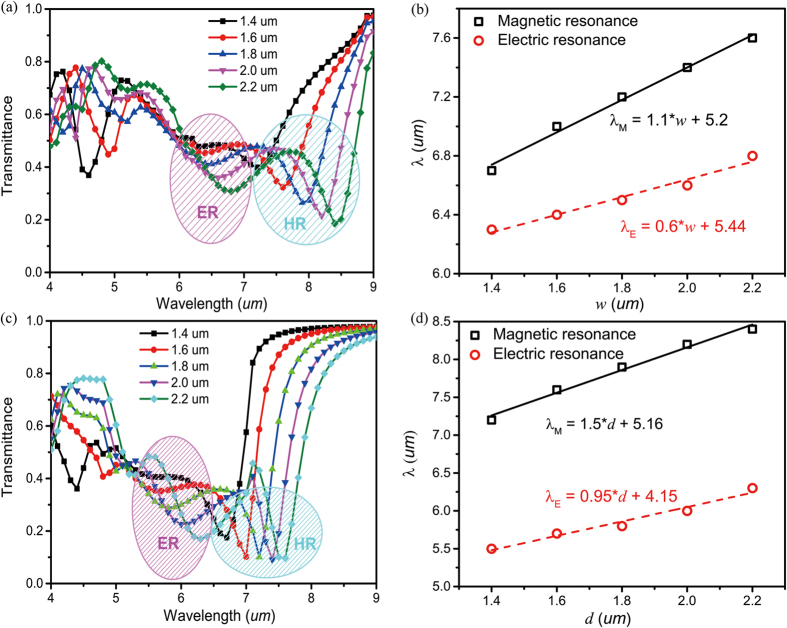
Dependences of the electric and magnetic resonant wavelengths on the geometry parameters. (**a**) Transmission spectra of Si metamaterial as a function of width w. (**b**) The wavelength dependence of magnetic resonance (MR, λ_M_) and electric resonance (ER, λ_E_) for different w. (**c**) Transmission spectra of Si metamaterial as a function of height. (**d**) The wavelength dependence of magnetic resonance (MR, λ_M_) and electric resonance (ER, λ_E_) for different d.

According to Mie resonance theory for dielectric particles^29^, each dielectric particle is equivalent to a magnetic dipole near the first resonant mode with longer wavelength and to an electric dipole near the second resonance mode with shorter wavelength. To further determine the origin of these two resonance modes in our design, the dynamic electric and magnetic field and displacement current distribution inside the Si cuboid are calculated and shown in Fig. 2(b–e). Consistent with previous work on Te metamaterials^16^, the electric and magnetic fields are mainly localized inside the Si cuboid at both resonant wavelengths. However, because of the existence of the BaF_2_ substrate, most of the electric and magnetic field distributions are not uniform and concentrated on the bottom of the cuboid. The displacement current in the x-z plane is greatly enhanced [Fig. 2(b)] at 8.06 μm and shows a typical loop surrounding the cuboid, whereas the magnetic field [Fig. 2(c)] plotted at the same wavelength in the y-z plane shows confinement in the center of the cuboid. This mode is corresponding to the TE_011_ mode of the Mie resonance and working as the magnetic activity resulted from the enhancement of the displacement current inside each rectangular, which gives rise to a macroscopic bulk magnetization of the composite^30^. At the second Mie resonance of 6.46 μm, the linearly polarized displacement current along x axis inside the cubes is greatly enhanced, giving a resonance electric field pattern on x-z plane similar to electric dipole characteristic [Fig. 3(d)]. Meanwhile, the magnetic field distribution in y-z plane shows obvious surrounding pattern although the BaF_2_ substrate seriously affects the symmetry of the pattern. These correspond to the TM_011_ mode of the Mie resonance and lead to electric dipole behavior at far field^31^.

After exploring the underlining mechanism through the near-field profiles, the Si-based metamaterial has been further examined by changing the cuboid geometries. Since the coupling between neighboring cuboids will weaken both the electric and magnetic resonances, the period p is selected to be more than twice of the width w. Figure 3(a) shows the transmission of the normally incident TM plane waves as a function of the width w varying from 1.4 to 2.2 μm with a step of 0.2 μm The height d is kept as 1.9 μm and periodicity as 6 μm. The amplitudes of both electric and magnetic resonances are strengthened with the increase of w. The transmission deep of magnetic resonance decreases from 0.18 to 0.10 and the resonant wavelength (λ_M_) red-shifts from 6.75 μm to 7.75 μm. The wavelength shifts because that the displacement current loop covers a larger area in y-z plane for larger w. Similar phenomena hold true for TE polarization. The relationship between w and the wavelengths of magnetic resonance (MR) and electric resonance (ER) can be clearly seen in Fig. 3(b), where near-linear relationships between λ_M_, λ_E_ and w can be observed. Due to different field modes, the magnetic resonance and electric resonance show different slopes. Meanwhile, the dependences of electric and magnetic resonances on the cuboid height d have also been analyzed. Here we fix p = 6 μm and w = 2 μm. With the increase of d from 1.4 to 2.2 μm, the intensities of both the electric and magnetic resonance dipoles increase, indicating stronger Mie scattering effect. Similarly, a near-linear relationship between λ_M_, λ_E_ and d can also be observed (see Fig. 3(d)). We note that no saturation and no-linear behaviors have been observed in Fig. 3(b–d). This is because that the values of *p*/w and *p*/d are already larger than 2, and the coupling effect for both electric and magnetic resonances between neighboring Si cuboid is negligible.

**Figure 4:**
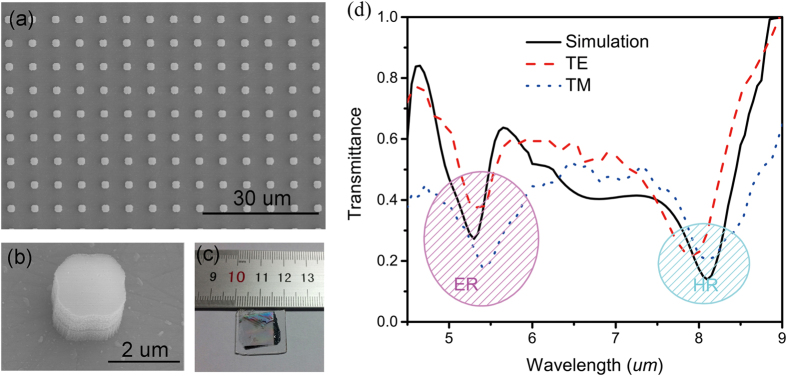
(**a**) SEM image of the final metamaterial structure consisting of an array of Si cuboids. The scale bar for the SEM image is 30 μm. (**b**) SEM image of a single Si resonator with a diameter of 2 μm. (**c**) Camera image of the large-scale pattern (∼2 cm × 2 cm) of Si metamaterial on a transparent BaF_2_ substrate. (**d**) The simulated and measured transmission spectra of the fabricated sample.

The fabrication process of the Si-based metamaterial began with a layer of α-Si (ε = 12.04, thickness = 1.7 μm) followed by wafer-scale patterning and reactive ion etching (RIE). First, a layer of α-Si with thickness of 2 μm is deposited onto a mid-infrared transparent barium fluoride (BaF_2_) substrate (2 × 2 cm^2^) with electron-beam evaporation. The deposition rates are kept at 1 Å/S with deposition temperature of 400 °C. Then a 2 μm photoresist (AZ2020) is spin-coated onto the α-Si layer and the patterns are transferred from photomask to photoresist via UV exposure and developed within AZ300 MIF. Due to the advantage of photolithography, the pattern can be easily fabricated in large scale without any defect. The resulting photoresist pattern is then used as a protective mask for a directive reactive-ion etching process. The whole sample is etched with SF_6_/C_4_F_8_ mixed gas in inductively coupled plasma (ICP, Oxford ICP180) with etching rate of 200Å/m. Then the sample is achieved by removing the remaining photoresist with acetone. The top-view scanning electron microscope (SEM, S4700, Hitachi) images of the final Si metamaterial and single typical Si cuboid are shown in Fig. 4(a,c). Square shape can be clearly observed for the single cuboid even though the detail shape is slightly detuned from the design due to the resolution limitation of photolithography. The width of the cuboid is around 2 μm and the period is 6 μm. These parameters are specified design in order to obtain strongest resonances by removing the electric and magnetic dipoles coupling effects.

The sample is then studied by measuring the transmission spectrum. Here, normally incident, polarized, broadband mid-IR light from a Nivolet380 Fourier Transform Infrared (FTIR) spectrometer is incident onto the surface of the sample (beam spot size of ∼2 mm) and the transmitted light from the back side of the sample is collected by an mercuric cadmium telluride (MCT) detector and normalized to that from a bare BaF_2_ substrate. The red dashed and blue point curves in Fig. 4(d) show the transmission spectra for TE and TM polarizations, respectively. Similar to the initial design, two resonances can be clearly observed in both of these spectra. And only slight difference can be seen between TE and TM polarized transmission spectra. This kind of difference is caused by the non-symmetry structure during fabrication.

The optical response of this fabricated Si cuboid arrays has also been matched by simulated spectra through importing the geometry parameters from the SEM image into numerical model. Since no difference between TE and TM polarizations in simulation, only simulated transmission curve for TM polarization are plot in Fig. 4(b). The simulated transmission spectrum also shows two resonances at 8.2 μm and 5.4 μm. The wavelength of simulated magnetic resonance (8.2 μm) agrees well with the experimentally observed feature (8.3 μm). And the simulated electric resonance at 5.3 μm also matches very well to the experimental results at of 5.4 μm (TM) and 5.35 μm(TM). The measured transmission (37% for TE polarization) is much larger than the simulated value (27%), indicating a much weaker electric resonance in experiment. This discrepancy arises from several reasons. Firstly, there is a capping layer formed during etching, which can decrease the effective refractive index of the whole cuboid. Secondly, the non-uniformity of the as-fabricated cube shape will further weaken the electric resonance and broad the resonance bandwidth as demonstrating in the transmission spectrum.

**Figure 5:**
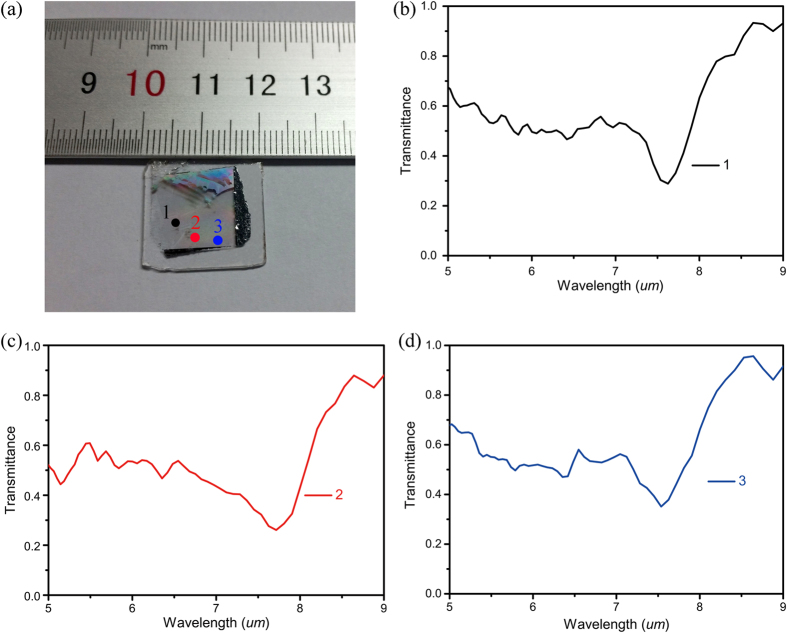
Transmission spectra for three points located away over a 2 × 2 cm^2^ area of the Si metamaterial.

To characterize the uniformity of the Si metamaterial over a large area, a spatial scanning of transmission spectrum on three positions over 2 × 2 cm^2^ area of the fabricated sample is carried out (Fig. 5(a)). The spatial transmission scan, shown in Fig. 5(b–d), indicates a very similar magnetic and electric resonances behavior with an average magnetic resonance wavelength on 7.6 μm with a largest deviation of only 0.1 μm, which is at the same level as the measurement resolution of 0.08 μm. The transmission intensity at magnetic resonance has an average number of 29.3% with a largest deviation of 5.7%. The uniformity of the transmission wavelength deep over such large areas indicates that the homogenized and defect free distribution of the Si cuboids benefiting from the standard and highly develop silicon devices fabrication method. The homogeneous properties of the metamaterial can be hold over even larger area size up to 12 inch since facilities and process in semiconductor field have been well developed to such large area. However, in order to further match the simulated results to the experimental data, the imaginary part of the silicon need to increase to as large as 0.02. The number is far higher than that for LPCVD or PECVD silicon and 2 orders larger than that for the crystalline silicon-on-insulator^32^. The huge loss of the structure can be from several aspects. First of all, the electron beam physical vapor deposition will result in non-conformal deposition of rough surfaces. Secondly, dry etching damage and contamination will affect the sidewall of the silicon cuboid. Finally, the capping layer formed during etching will strongly weaken the resonant behaviors. We anticipate that significantly lower-loss metamaterials can be achievable as we continue to remove the capping layer formed during etching, improve the quality of Si film and reduce the silicon absorption.

## Conclusion

In summary, we demonstrated that wafer scale silicon magnetic metamaterials can be fabricated using standard integrated circuit fabrication process. The defect-free Si-based metamaterial can overcome both of absorption loss and scalability limitations. Magnetic resonance is realized in mid-infrared wavelength at the typical position around 8 μm, which electric resonance can be obtained at 5.4 μm. The transmission spectra taken at different positions of the sample prove the uniformity of the magnetic response from the structure up to centimeter scale. The results further confirm the validity for scaling up fabrication of defect-free all-dielectric metamaterials to wafer scale using a simple, low-cost, and high-throughput method.

## Methods

To investigate the magnetic and electric activities, Si sub-wavelength cuboid arrays are studied under polarized incident light with commercial Finite Element Method (FEM) simulation software, COMSOL Multiphysics. The simulation considers a volume spanning of period p in x, y directions, and 40 μm in z direction around the cuboid. The Si cuboid is located at x = y = z = 0 and covered by air (optical index of 1). Barium fluoride (BaF_2_) is selected as the substrate due to its low refractive index (n_BaF2_ ~ 1.471) and transparent window up to 10 μm. All four boundaries of the computational volume in x and y axis are terminated with convolutional periodic boundary layers. The non-uniform grid resolution varies from 25 nm for areas at the periphery of the simulations to 5 nm for the region in the immediate vicinity of the cuboid (±300 nm in x and y directions and ±150 nm in z direction). The excitation of the metamaterial is made with a plane wave launched 40 μm above the structure from the air side, linearly polarized with electric field along the x-axis (noted as TM polarization) and propagating along the z-axis, as shown in Fig. 1.

## Additional Information

**How to cite this article**: Yi, N. *et al*. Large-Scale and Defect-Free Silicon Metamaterials with Magnetic Response. *Sci. Rep*. **6**, 25760; doi: 10.1038/srep25760 (2016).
